# Development of a Tailored HIV Prevention Intervention for Single Young Men Who Have Sex With Men Who Meet Partners Online: Protocol for the myDEx Project

**DOI:** 10.2196/resprot.7965

**Published:** 2017-07-19

**Authors:** Jose Arturo Bauermeister, Ryan C Tingler, Michele Demers, Gary W Harper

**Affiliations:** ^1^ University of Pennsylvania Philadelphia, PA United States; ^2^ University of Michigan Ann Arbor, MI United States

**Keywords:** eHealth, HIV prevention, Internet, risk reduction, sexually transmitted infections

## Abstract

**Background:**

New cases of human immunodeficiency virus (HIV) among young men who have sex with men (YMSM), aged 18 to 24, underscore the importance of developmentally-informed HIV programs for YMSM. We developed an online intervention focused on risk reduction strategies across different sexual partner types. Intervention activities focus on assisting YMSM reflect on their partner-seeking behaviors, develop sexual decision-making rules to reduce their HIV risks, and consider the adoption of HIV prevention behaviors.

**Objective:**

This pilot, randomized controlled trial (RCT) aims to examine the feasibility, acceptability, and preliminary efficacy of a tailored, Web-based HIV prevention intervention for single YMSM.

**Methods:**

We designed a prospective RCT of online-recruited cis-gender men (N=180) who reported recent unprotected anal intercourse, self-report as HIV negative or are unaware of their HIV status, and meet sexual partners through online dating apps. Individuals in the control arm receive an attention-control condition that includes HIV/sexually transmitted infection (STI) information currently available on sex education websites. Individuals in the intervention arm receive a 6-session Web-based program tailored on their demographic information, partner-seeking behaviors and relationship desires, and prior sexual attitudes and behaviors. This tailored content will match HIV prevention messages and safer sex skills with YMSM’s outcome expectancies when meeting new partners and thereby help them consider how to integrate safer sex practices into different partner types. Study assessments are taken at baseline, 30-, 60-, and 90-day follow-ups. Intervention acceptability and preliminary efficacy will be explored in sexual risk behaviors and HIV/STI testing.

**Results:**

The RCT launched in November 2016 and is ongoing. To date, 180 eligible individuals have been enrolled, consented, and randomized. Of the 120 individuals in the intervention arm, 51.7% (62/120) identify as non-Hispanic white and half of the control arm identifies as non-Hispanic white. There were no differences observed by arm for race and/or ethnicity, age, or sexual orientation.

**Conclusions:**

Although there are in-person evidence-based interventions with proven efficacy for YMSM, few HIV/STI prevention interventions delivered online exist. Online interventions may ease access to comprehensive HIV/STI education among YMSM and allow personalized content to be delivered. The online intervention that we developed, myDEx, aims to alleviate the gaps within HIV prevention for YMSM by utilizing tailored, Web-based content with the goal of developing skills for same-sex dating and relationship building, while reducing their risks for HIV/STI.

**Trial Registration:**

ClinicalTrials.gov NCT02842060; https://clinicaltrials.gov/ct2/show/NCT02842060 (Archived by WebCite at http://www.webcitation.org/6rcJdxF9v)

## Introduction

### Scientific Background

Interventions specific to young men who have sex with men (YMSM) are needed to curtail the rise of new *human immunodeficiency virus* (HIV)/acquired immunodeficiency syndrome (AIDS) infections. In 2015, in the United States, YMSM aged 13 to 24 years had the greatest percentage increase (87%) in diagnosed HIV infections [1], with black and Latino YMSM accounting for the greatest proportion of new infections among men who have sex with men (MSM) [2]. HIV prevention tools that are culturally and developmentally adapted for this population are needed [3]. To this end, we developed an online intervention that promotes HIV prevention behaviors (eg, condom use, pre-exposure prophylaxis (PrEP) awareness and uptake, HIV/sexually transmitted infection [STI] testing) and reduction of risk behaviors (eg, number of sexual acts where HIV transmission could be possible) for single YMSM presumed to be HIV-negative who engage in unprotected (ie, condomless) anal intercourse (UAI) with sexual partners met online.

Most individuals explore and integrate aspects of their sexuality into their personal identity as they transition from adolescence into young adulthood (ie, 15 to 24 years of age). Serial dating and involvement in different types of relationships has been documented as helping youth to define their sexual identity, to narrow the characteristics sought in long-term relationships, and to practice safer sex negotiation skills [4]. However, compared to heterosexual counterparts, YMSM may not readily receive support and advice from family, peer, and school systems on how to date and seek out same-sex partners. Furthermore, YMSM do not receive instruction on how to have anal sex as part of their sex education [5-7] or guidance on how to negotiate condom use with their partners [8]. This is particularly problematic as YMSM’s participation in dating behaviors during this period coincides with their mean age of initiating anal sex [5,6,8-10], and may create unique HIV vulnerabilities as they engage in partner-seeking behaviors. Recognizing the importance of relationship pursuits in this period, we developed a comprehensive sex education intervention that addresses HIV risk reduction in the context of same-sex dating and safer sex negotiation activities with sexual partners.

### Objectives

HIV prevention interventions for single YMSM who meet partners online must account for different relationship typologies common in this developmental period: romantic interests, casual encounters/hook-ups, and friends with benefits [11]. Although MSM couples-based intervention projects are underway [12], these interventions may not be translatable to single YMSM who, by definition, are not in a relationship and do not have a “main partner.” This pilot, randomized control trial (RCT) aims to examine the feasibility, acceptability, and preliminary efficacy of a tailored, Web-based HIV prevention intervention for single YMSM called myDEx. The pilot RCT compares our tailored intervention to an attention-control condition. Using a 2:1 block randomization, we examine our intervention’s feasibility and acceptability among 180 single, HIV-negative YMSM (50% racial/ethnic minorities), and gather preliminary behavioral data to inform a future efficacy trial. Assessments are collected at baseline, 30-, 60-, and 90-day follow-up.

## Methods

### Trial Design

The research activities involve a prospective, pilot RCT of approximately 180 online-recruited cis-gendered MSM. Individuals in the experimental arm receive a 6-session, Web-based program with interactive content using story-telling, case scenarios, risk reduction strategies, and graphics and/or videos. Individuals in the control arm receive a 6-session online, non-tailored HIV prevention intervention (NTHP) using information available on the Centers for Disease Control and Prevention website. The NTHP condition will have an interface similar to myDEx aesthetics and site features to avoid confounding due to site design and navigation.

**Figure 1:**
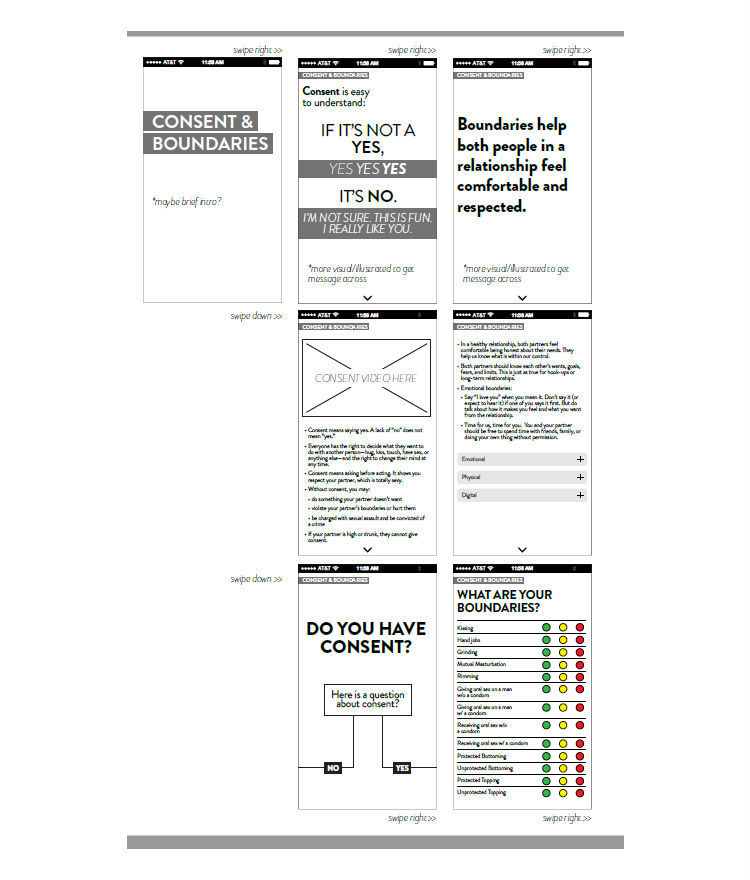
Wireframe example used during the development of myDEx.

A youth advisory board (YAB) was recruited for this study. YAB members (N=3) were YMSM between the ages of 18 and 24 and diverse across race and/or ethnicity, educational attainment, socioeconomic status, faith, and urban/rural residential background. YAB members were hired as part-time research assistants. The YAB’s roles and responsibilities included (1) providing input into the proposed intervention content; (2) brainstorming with the research team on how to deliver the content using active learning and youth-friendly engagement; and (3) leading or co-facilitating trainings for the WebApp developers to learn about same-sex attractions and dating behaviors and popular MSM-specific apps used for dating and hooking up. As each intervention session was developed, the YAB and research team independently brainstormed what content and activities could be included in each session. The ideas were then discussed as a team, ordered by relevance for the session and within the session, and annotated for the developers to consider while building the wireframes (Figure 1). These discussions served to inform the user navigation of our intervention, including how to organize content within sessions into 3 levels focused on a core message (level 1), in-depth discussion of topics linked to that message (level 2), and an activity component (level 3).

Our intervention will be pilot tested using a racial and/or ethnically diverse sample (50% racial/ethnic minority) of single YMSM living throughout the United States (N=120), using an attention-control comparison condition (N=60) to test our intervention’s feasibility, acceptability, and preliminary efficacy. Primary outcomes of interest include increased consistent condom use across partner types and HIV/STI testing, and decreased UAI occasions and partners. Participants will complete 3 online follow-up assessments at 30, 60, and 90 days, each lasting approximately 30 minutes.

### Eligibility Criteria

Eligible participants are (1) male at birth and identify as male; (2) between the ages of 18 and 24 (inclusive); (3) self-report as single; (4) self-report as HIV-negative or are unaware of their HIV status; (5) speak and read English; (5) report using online dating apps; and (6) report UAI with a male partner in prior 6 months. Participants for the trial are from across the United States, with recruitment via online advertisements placed on popular social and sexual networking sites. Promotional materials describe the study and provide a link to a page containing basic study information, including a short description of study activities.

Potential participants interested in the study complete a short eligibility screener. If eligible, participants complete an online consent form. Individuals who do not meet eligibility criteria or do not consent into the study (if eligible) are thanked for their time and are exited from the study site. Once consent is obtained, participants complete a 30-minute baseline questionnaire online. As part of the baseline questionnaire, participants provide information that may help us contact them for follow-up assessments and verify that they are not fraudulent or duplicate entries [13,14]. We use best practices [15,16] to retain participants (eg, comprehensive locator information that includes participants' cell phone number, email, Facebook username, etc). When the baseline survey is complete, participants are randomized into the 2 study arms.

### Incentives

Individual participants each receive US $30, $15, $20, and $25 for completing the baseline assessment and the 30-, 60-, and 90-day follow-ups, respectively. The incentives are back loaded to encourage completion of all 3 data collection time points and reduce participant attrition over time.

### Sample Size

The goal is to enroll and maintain a sample of 180 single YMSM over a 3-month study period, with an expected retention rate of 80%. As a pilot, we recognize that we may not have sufficient power to detect small effect sizes; however, this design will inform our subsequent large-scale RCT, allowing us to identify and address implementation and retention challenges that could arise in the larger trial. Specifically, the primary purposes of this pilot trial are (1) to demonstrate the feasibility of the methods proposed for a subsequent trial; (2) to stabilize procedures that are replicable; and (3) to determine important parameters with sufficient accuracy to reliably estimate sample size and power for a future RCT. As a result, we are not powered to estimate small effect sizes or carry out sophisticated statistical analyses; rather, we seek to estimate key study parameters with sample means and proportions together with 2-sided 95% CIs, and test the primary null hypotheses at the traditional 2-sided level alpha of .05.

We will have 80% power to detect a medium intervention effect (Cohen d less than .35) at alpha of .05 in a continuous measure using a repeated measures group design (N=180) with 4 observations (baseline and 3 follow-up assessments) when the standard deviation (SD) is 1 and the correlation between observations on the same subject (rho) is .6. For dichotomous outcomes, we estimate 80% power at an alpha of .05 to detect an odds ratio (OR) of 2.11 or greater assuming that the proportion for the intervention condition is .58 and the attention-control condition is .40, with rho also being .60. We note that there is some controversy regarding the use of “pilot data” to estimate actual effect size, as specified by Kraemer et al [17]. We agree with their view that clinically meaningful effect sizes should be decided based on extrinsic, clinical judgment grounds, and not based on the pilot data which are too few, typically, to obtain reliable conclusions. However, we can use these data to rule out unusually large or small effects through standard 95% CI procedures. Thus, we will confirm that extrinsic effect sizes are contained within our CIs. Even with 20% attrition, we will have preliminary effects with adequate error margins to inform the subsequent trial.

### Randomization

Individuals are randomized to either the intervention arm (myDEx) or the attention-control arm (NTHP) using a stratified 2:1 block randomization design. Block randomization is stratified by race (eg, white versus non-white), with equal allocation in each group. Treatment assignments are generated with the use of a pseudo-random-number generator with permutated blocks that are used to ensure balance across participants’ assigned condition.

### Theoretical Framework

A dual processing, cognitive-emotional decision making framework [18] informs our intervention framework. Decision-making researchers have noted that affective motivations may be processed more rapidly than cognitive motivations and may result in decision-making that is affectively motivated rather than analytically motivated [19].

To increase participants’ cognitive motivations, our intervention is informed by the Integrated Behavior Model (IBM), one of the leading behavior change theories in HIV/STI research given its adaptability across populations and its application in health communication research [20]. Content geared to increasing cognitive motivations will focus on risk reduction attitudes, norms, and perceived behavioral control. Positive attitudes include assurance of avoiding STIs, increased control, responsible decision-making, and prolonged and enjoyable sexual encounters. Negative attitudes include discomfort, decreased sensitivity and ineffectiveness in preventing STIs, interrupting the mood, and assuming sickness or irresponsible and/or immoral behavior. We will also address both descriptive norms (ie, perceived prevalence of behaviors in YMSM’s social network) and personal norms (ie, anticipated regret), as YMSM’s norms may be highly influential on individual attitudes during this developmental period. Finally, we also address YMSM’s perceived behavioral control, recognizing that their ability to engage in risk reduction behaviors may vary across partner types (eg, romantic interest, casual partner, friends with benefits).

**Figure 2:**
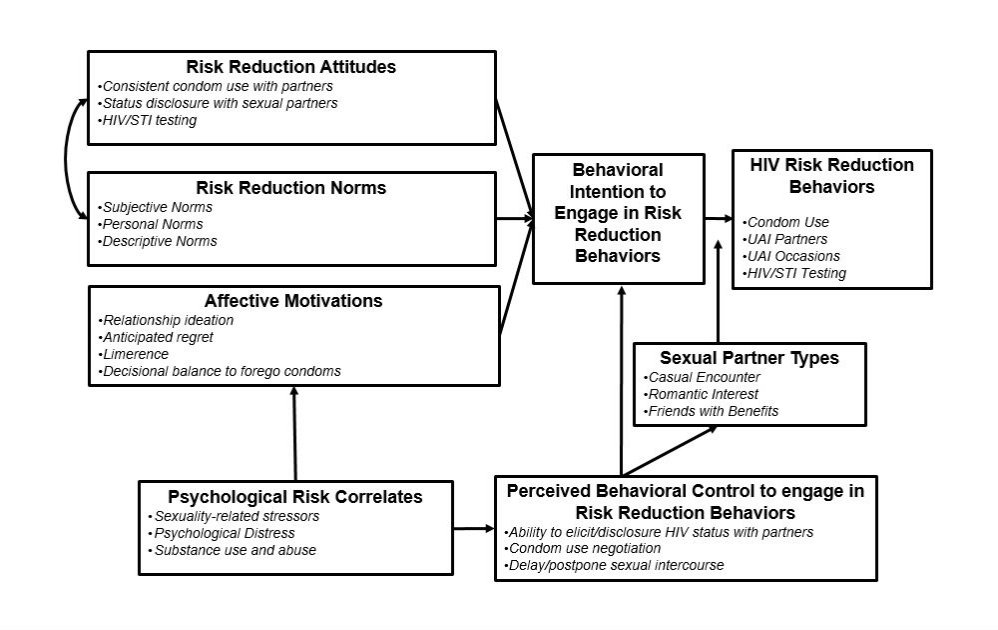
Conceptual model informing the myDEx intervention.

Although IBM has invaluable strengths, individuals with conflicting affective and cognitive motivations report less correspondence between their intentions and behavior [21-23]. Consequently, intervention also acknowledges that YMSM’s affective motivations may be health promotive (eg, relationship ideation) or risk enabling (eg, limerence). Building on our prior work, we hypothesize that YMSM reporting greater relationship ideation [24] will report fewer HIV/AIDS risk behaviors (ie, health promotive affective motivations). In addition, we include anticipated regret [25,26] (ie, anticipation of an emotional reaction following an unintended behavior) as a health promotive construct, as it has been associated with fewer risk-taking behaviors among MSM [27]. However, we also hypothesize that YMSM who experience greater limerence [28] and who believe that foregoing condoms with their partners will create intimacy, love, and trust (decisional balance) [28,29] may place stronger value on being in a relationship and, in turn, fuel HIV/AIDS risk behaviors (eg, more UAI partners and occasions). Therefore, in our intervention sessions, we address how affective motivations may influence YMSM’s decision-making regarding consistent condom use, UAI partnerships, and HIV/STI testing behavior. Finally, the model acknowledges that YMSM’s cognitive and affective motivations may be influenced by YMSM’s sexuality-related stressors (eg, internalized homophobia), psychological distress (eg, depression, anxiety, loneliness, low self-esteem), and substance use and abuse [10]. These risk correlates may influence YMSM’s ability to regulate their affective motivations and to engage in risk reduction behaviors due to limited behavioral control (eg, impairment due to being drunk or high). In sum, as shown in Figure 2, this cognitive-affective decision-making model offers a strong, theoretically-driven foundation to inform our intervention.

### Intervention Arm

The proposed intervention consists of a 6-session, Web-based program (myDEx). Each session’s modular content is delivered through interactive, tailored story-telling, case scenarios, motivational interviewing strategies, and graphics and videos. Cognizant of challenges maintaining users’ attention in a Web app and to facilitate delivery through a mobile phone with app capabilities, we designed each session to keep users engaged for 10 minutes. Tailored content maximizes content persuasiveness and relevance and facilitates behavior change by enhancing message processing and message impact through personalization, content matching, and feedback. Personalization increases a user’s attention to the message by raising awareness to customization of content (eg, “Based on your answers…”) and making it more meaningful (eg, refer to the participants’ behaviors, match photographs to age group and race/ethnicity). Content matching is a way to target factors known to influence behavior change by providing users with relevant information designed to support positive behaviors. Feedback about participants’ answers increases attention and impact through self-referential thinking, comparative feedback (eg, “Compared to others...”) to validate positive beliefs and adjust errors in normative beliefs, and provides evaluative opportunities linked to individuals’ underlying values and motivations.

Within each session, participants have access to brief activities designed to build their HIV risk reduction skills and promote self-reflection about YMSM’s sexual health and partner-seeking behaviors. Interactive activities include (1) role-play scenarios regarding condom use negotiation; (2) a diary to log their dating experiences throughout the study; (3) quizzes regarding their ideal relationships, including short-term and long-term relationships; and (4) opportunities for users to develop dating strategies. Online modules and activities can be repeated so that YMSM may compare whether their answers are consistent with the tailored suggestions and revisit content and/or revise their answers to reinforce the material.

**Figure 3:**
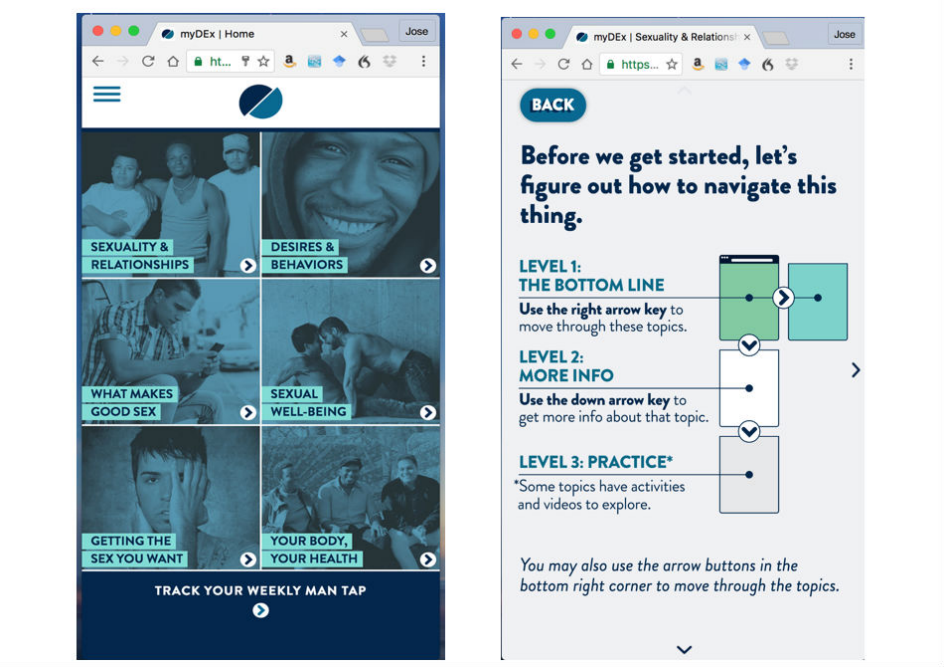
myDEx home screen and navigation instructions.

Participants must complete the first session before they can access the other 5 sessions and interactive features. Session 1 (“Sexuality & Relationships”) serves as an introduction and focuses on the importance of feeling comfortable talking about sexuality, relationship desires, and health. This session focuses on acknowledging and normalizing YMSM’s affective motivations and foreshadowing where participants can learn more about different topics of interest within the remaining 5 sessions of the intervention. Session 2 (“Desires & Behaviors”) transitions into a discussion regarding different relationship types (eg, romantic relationships, friends with benefits, hookups) and sexual decision-making. It highlights the importance of knowing what kind of relationship one desires, in both the short-term and long-term, and the role of sex in exploring these relationships with different types of partners. Session 3 (“What makes good sex”) provides a comprehensive sex education review focused on same-sex behaviors, including the importance of sex positivity, varying sexual practices, and sexual consent. Session 4 (“Sexual well-being”) reinforces how to reduce HIV and STI risks when engaging in anal sex, including clarification on what lubricants and condoms are best suited for anal intercourse, facts about HIV and STI transmission, and the importance of status disclosure prior to sex. Session 5 (“Getting the sex you want”) provides opportunities for YMSM to learn strategies to improve their sexual communication with partners before, during, and after sex. This session includes how to discuss HIV testing history and status awareness with prospective partners online, how to ensure their physical safety when meeting a new partner, and the value of discussing condoms and PrEP with partners. Session 6 (“Your body, your health”) summarizes key messages from prior modules and offer HIV/STI testing resources and PrEP locations in their area. The main menu and final navigation instructions for the intervention is shown in Figure 3.

### Control Arm

We created a 6-session, Web-based attention-control comparison to match myDEx in time and attention yet containing have non-tailored and non-interactive content (NTHP). Session 1 (“What is HIV?”) focuses on an introduction to HIV, including how it is transmitted and what can increase a person’s risk of acquiring it. Session 2 (“HIV and the LGBTQ community”) focuses on raising awareness of the HIV prevalence and incidence among YMSM in the United States. Session 3 (“HIV Risk”) focuses on behavioral strategies that can be used to reduce HIV risk. Session 4 (“Sex & HIV”) describes the HIV risks associated with oral and anal sex. Session 5 (“What about condoms & lube?”) focuses on the importance of condoms and lubricant. Session 6 (“What about HIV medications?” *)* presents an overview of PrEP.

**Figure 4:**
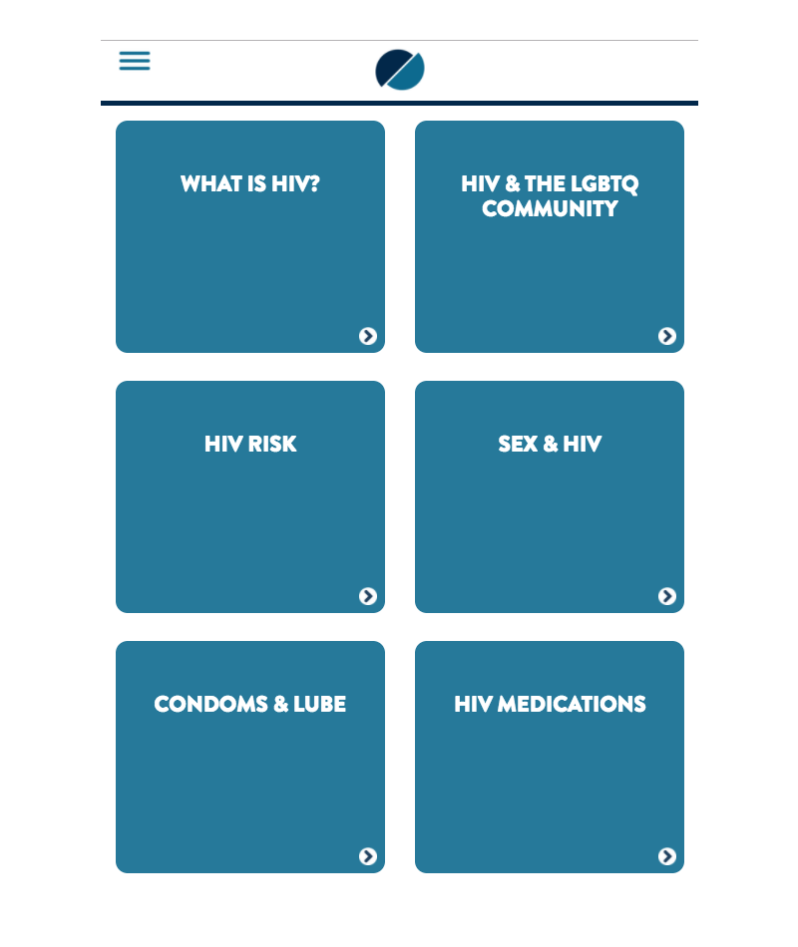
Home screen of the control condition.

Our attention-control condition allows us to avoid confounding due to content (ie, comparing myDEx to a non-HIV “health promotion” intervention) and ensures that all YMSM receive some HIV prevention content given their high vulnerability to HIV (Figure 4). Further, this comparison will help us critically examine the extent to which tailoring increases YMSM’s acceptability to the program, beyond having a non-tailored, non-interactive intervention. We acknowledge that the comparison condition will make it harder to detect an intervention effect in our outcome assessments; however, our pilot trial’s primary goal is to test the intervention’s feasibility and acceptability, and subsequently estimate critical parameters that may be required for adequate power estimation in a subsequent large-scale RCT trial.

### Feasibility and Acceptability Outcomes

The study assesses feasibility by examining (1) time to recruit 180 YMSM to the intervention; and (2) retention of rate across study arms. Intervention acceptability data are collected at the 30-day follow-up assessment. We use the following different assessments: (1) self-intervention evaluation form (SEF) [30]; and (2) Client Satisfaction Questionnaire (CSQ-8) [31]. The SEF is a brief, 13-item questionnaire that elicits information about the participant’s experience with the intervention (ie, was the intervention interesting, was it relevant to their life, did they learn from the intervention). The CSQ-8 is used at the completion of the intervention and at the 30-day and 90-day follow-up surveys to assess YMSM’s satisfaction with the intervention, including the content, site layout and design, and general satisfaction. These domains are assessed on a 4-point response scale with individually-specified anchors. The CSQ-8 has demonstrated high internal consistency across a large number of studies [32]. The SEF and CSQ-8 take approximately 10 minutes to complete.

We also track users’ actions in the intervention as process evaluation data. These data include the number of times they visit the site, their geographic location and the website from which they linked to our site, time spent in each session, number of times a user returns to a session, and which interactive features were “clicked on” during sessions. In addition, we ascertain participants’ acceptability and satisfaction of each session after it is viewed using the SEF.

### Behavioral Outcomes

We measure the change in number of risky sexual partnerships and change in HIV testing behavior as our primary outcome measures via the baseline, 30-, 60-, and 90-day online surveys.

#### Sexual Behaviors

We use the Sexual Practices Assessment Schedule (SPAS) [33] to quantify the number of occasions of different sexual acts (oral, anal, receptive, and insertive) with different partner types. SPAS allows us to estimate the number of unprotected anal intercourse partners and occasions across partner types, as well as the proportion of instances when condoms were not used [28,34,35]. SPAS also ascertains YMSM’s use of condoms during the past 30 days, whether they knew whether their partners were on PrEP, and whether they knew their partners’ HIV status prior to sex.

#### HIV/STI Testing Behaviors

We ask YMSM to indicate the date of their most recent HIV and STI tests. Subsequently, we ask participants to note if they have received a medical diagnosis as having one or more STIs in their lifetime and the date of their most recent STI test if available. In each follow-up survey, we ask participants whether they have gone to get tested for HIV and/or STIs in the prior 30 days. If tested, participants are asked to indicate what tests they received and whether they had been medically diagnosed as having HIV or a STI. At follow-up, we ask YMSM whether they have had any changes in their HIV status. Newly diagnosed cases are asked if they were linked to care.

Secondary measures are also being measured in our study.

#### Motivations to Engage in HIV Prevention Behaviors

We measure YMSM’s attitudes, subjective norms, and self-efficacy using previously tested scales with MSM. We use existing items measuring condom use intentions with different partner types and self-efficacy to negotiate condoms with different partner types [36-39]. We also measure PrEP awareness, uptake, and adherence during the study.

#### Substance Use Prior To or During Sex

We assess alcohol, tobacco, and other drugs (ATOD) use over the past 30 days. We then assess the use of alcohol and/or illicit drug use prior or during sex.

#### Psychological Well-Being

We measure depression and anxiety symptoms as markers of psychological distress. Depression (6 items) and anxiety (6 items) symptoms in the past week are measured using the Brief Symptom Inventory [40].

### Statistical Analysis

Prior to conducting multivariable analyses, we examine study variables using descriptive statistics and test for differences across demographic characteristics (eg, race and/or ethnicity, age, education) using *t* tests, analysis of variance (ANOVA), and chi-square analysis, as appropriate. Systematic baseline differences are not expected due to randomization; however, in the event that some parameters differ across conditions at baseline, they will be included as covariates in subsequent analyses. We calculate descriptive summary statistics corresponding to the study variables at each visit to understand any temporal patterns, as well as compare the 2 treatment groups in terms of average change from baseline to post-intervention.

### Trial Registration, Ethics, Consent, and Institutional Board Approval

The research and ethics presented in this study have been reviewed and approved by the University of Michigan Institutional Review Board (HUM00091627). The University of Pennsylvania ceded regulatory oversight to the University of Michigan. The study is also registered on ClinicalTrials.gov (NCT02842060).

## Results

myDEx was launched in November 2016 and is ongoing. Initially, 23,365 individuals visited the study site and 1230 were screened for eligibility. Of the 392 eligible participants, some did not start the baseline survey (3.8%, 15/392) or did not finish the baseline survey (7.9%, 31/392). Of the 263 participants who completed the baseline survey, 31.6% (83/263) were disqualified due to having duplicate accounts or having falsified their identity (eg, cis-gender women).

**Figure 5:**
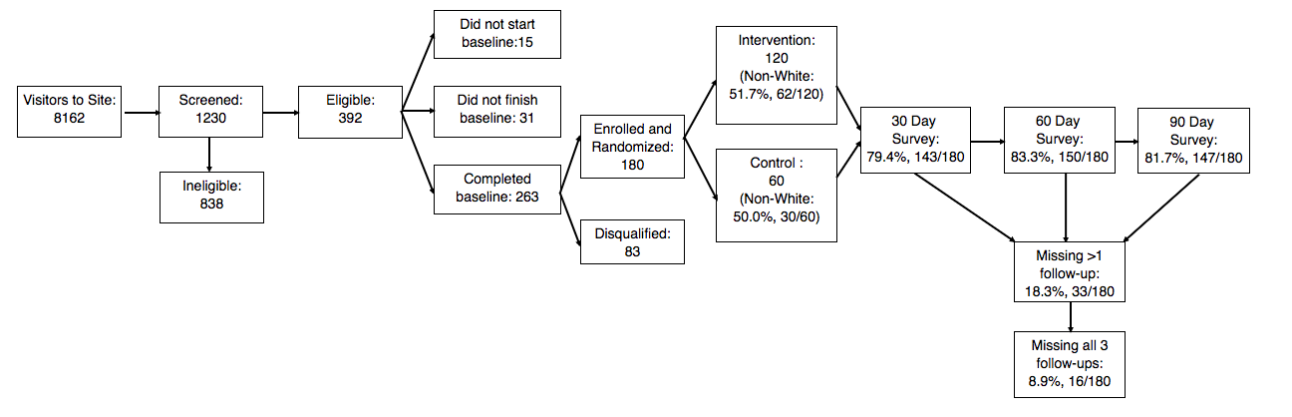
Recruitment and retention of myDEx participants.

We enrolled and randomized 180 YMSM into the trial (Figure 5). The majority of the participants are white (66.7%, 120/180), followed by multiracial (16.1%, 30/180), black (10.0%, 18/180), Asian (5.6%, 10/180), and Middle Eastern (0.6%, 1/120) or Native American (0.6%, 1/120). In addition, 30.0% (54/180) of the sample reported being Hispanic/Latino. Of the 120 individuals in the intervention arm, 51.7% (62/120) identify as non-Hispanic white; 50.0% (30/60) of the control arm identify as Non-Hispanic white. No differences are observed by arm for race and/or ethnicity (χ^2^_4_ = 0.53; *P*=.97). The mean age of participants is 21.67 (SD 1.81), with no differences observed by arm (t_(179)_ = 1.05; *P*=.30). The majority of participants identify as gay (88.3%, 159/180) followed by bisexual (7.8%, 14/180) and queer (3.9%, 7/180). There are no differences between treatment arms (χ^2^_2_= 1.40; *P*=.50).

The follow-up assessments maintained high retention rates. The 30-day follow-up had a response rate of 79.4% (143/180). The 60-day follow-up had a response rate of 83.3% (150/180). The current 90-day response rate is 81.7% (147/180). Overall, we have at least 1 follow-up assessment for all study participants (ie, 9% of sample has not completed any follow-up assessment). Retention rates do not vary between treatment arms.

As we compared trial paradata to participants’ self-reported behavioral surveys, we discovered that 25 (41%, 25/60) of the control participants inadvertently accessed the intervention content through a programming error in the automated reminder emails meant to encourage continued engagement with the site. Given the cross-arm contamination, we excluded these control cases from future trial analyses between arms (N=35 control; N=120 intervention). The 25 excluded cases do not affect our randomization; we observe no sociodemographic differences between the revised control arm and intervention arm across age (*t*_153_ = –.80; *P*=.43), racial/ethnic minority status (χ^2^_1_= .10; *P*=.75; N=155), educational attainment (*t*_153_= –1.24; *P*=.22), or sexual orientation (χ^2^_2_ = 3.23; *P*=.20; N=155). Retention rates also do not vary.

Participant recruitment for myDEx is complete. Trial data are currently being analyzed and will be completed in mid-2018.

## Discussion

### Principal Findings

Given YMSM’s self-reported desire to access comprehensive sexual education through the Internet [41,42], often ranking the Web as their top resource to explore their sexuality, learn about MSM behavior, and refine their interests [41-44], designing and testing online HIV prevention interventions may present a number of advantages to reach and address the needs of YMSM. Online interventions can deliver tailored content to each user’s HIV risk behaviors and context, be accessed conveniently by a participant, presented across various platforms (eg., mobile phones, tablets, laptops), and reduce reach and accessibility issues due to geography and/or socioeconomic barriers. Furthermore, online delivered content can be standardized, ensuring higher intervention fidelity, and be presented through interactive features. This protocol may serve to address YMSM’s needs and offer insights on how to reduce their risk for HIV infection when seeking partners online.

### Limitations

We did not include biological confirmation of HIV/STI status through serologic tests. At this early stage of intervention development and testing, the added cost of biological testing is not warranted. HIV testing will be included in a full-scale efficacy trial. We will not be able to use the 25 excluded control cases due to contamination. This is less of a concern to this pilot trial as our protocol aims do not seek to detect differences in this trial. Moving forward, we urge scholars to examine paradata files alongside their survey data to reduce the potential of unintentionally contamination due to unforeseen programming errors.

### Conclusion

myDEx provides an opportunity to develop a culturally relevant evidence-based intervention for YMSM. Although there are in-person evidence-based interventions with proven efficacy, few HIV/STI prevention interventions delivered online exist for YMSM. Online interventions may ease access to comprehensive HIV/STI education among YMSM and allow personalized content to be delivered. myDEx aims to alleviate the gaps within HIV prevention for YMSM by utilizing tailored, Web-based content with the goal of developing skills for same-sex dating and relationship building, while reducing their risks for HIV/STI.

## Abbreviations

AIDS: acquired immunodeficiency syndrome
CSQ: Client Satisfaction Questionnaire
HIV: human immunodeficiency virus
IBM: Integrated Behavior Model
LGBTQ: lesbian, gay, bisexual, trans, and queer
MSM: men who have sex with men
NTHP: non-tailored HIV prevention
PrEP: pre-exposure prophylaxis
RCT: randomized controlled trial
SEF: self-intervention evaluation forms
SPAS: Sexual Practices Assessment Schedule
UAI: unprotected anal intercourse
YAB: youth advisory board
YMSM: young men who have sex with men
